# Multiple Pathways Suppress Non-Allelic Homologous Recombination during Meiosis in *Saccharomyces cerevisiae*

**DOI:** 10.1371/journal.pone.0063144

**Published:** 2013-04-30

**Authors:** Miki Shinohara, Akira Shinohara

**Affiliations:** 1 Division of Integrated Protein Functions, Institute for Protein Research, Osaka University, Suita, Osaka, Japan; 2 Department of Biological Sciences, Graduate School of Science, Osaka University, Suita, Osaka, Japan; University of Minnesota, United States of America

## Abstract

Recombination during meiosis in the form of crossover events promotes the segregation of homologous chromosomes by providing the only physical linkage between these chromosomes. Recombination occurs not only between allelic sites but also between non-allelic (ectopic) sites. Ectopic recombination is often suppressed to prevent non-productive linkages. In this study, we examined the effects of various mutations in genes involved in meiotic recombination on ectopic recombination during meiosis. *RAD24*, a DNA damage checkpoint clamp-loader gene, suppressed ectopic recombination in wild type in the same pathway as *RAD51*. In the absence of *RAD24*, a meiosis-specific *recA* homolog, *DMC1*, suppressed the recombination. Homology search and strand exchange in ectopic recombination occurred when either the *RAD51* or the *DMC1 recA* homolog was absent, but was promoted by *RAD52*. Unexpectedly, the *zip1* mutant, which is defective in chromosome synapsis, showed a decrease, rather than an increase, in ectopic recombination. Our results provide evidence for two types of ectopic recombination: one that occurs in wild-type cells and a second that occurs predominantly when the checkpoint pathway is inactivated.

## Introduction

Meiotic recombination ensures the segregation of homologous chromosomes at meiosis I by providing a physical linkage between them. Although this recombination generates both crossover (CO) and non-crossover (NCO) recombination products, only COs generate physical linkages between homologs known as chiasmata [1], [2]. Recombination during meiosis occurs predominantly between homologous chromosomes (inter-homolog recombination bias), although it has been reported that recombination between sister chromatids also occurs at reasonable frequency [3], [4]. On the other hand, during mitosis, recombination preferentially takes place between sister chromatids [5]. In addition, in some cases, recombination also promotes exchange between non-allelic (ectopic) sites on chromosomes. This non-allelic homologous recombination (NAHR) may result in chromosome translocations, deletions or inversions, which have been associated with instability of the genome [4].

Meiotic CO formation is initiated by the generation of double-strand breaks (DSBs) [6]. After nucleolytic processing of DSB ends, exposed single-stranded DNA is used by the recombination machinery to search for corresponding DNA sequences on a homologous chromosome (as opposed to a sister chromatid). One end of each DSB site is believed to engage in direct interaction with homologous sequences, whereas the other end does not participate in the initial homology search but is involved later in a step called “second-end capture” [7]–[9]. After identifying the complementary DNA sequence on the homolog, single-stranded DNA is thought to form an unstable D-loop. If a DNA strand synthesized from the invading 3′-end is dissociated from the template strand in the D-loop, it leads to the formation of NCO products through a synthesis-dependent strand-annealing pathway [7], [10], [11]. Alternatively, if the synthesized strand is not dissociated, the D-loop may be converted into a “stable” single-stranded invasion intermediate (SEI) [7]. Further processing leads to the formation of intermediates with two Holliday junctions, referred to as a double-Holliday junction (dHJ) [12], that are specifically resolved into reciprocal CO products [7], [10], [13].

In meiotic recombination, two RecA homologs, Rad51 and Dmc1, play a critical role in the homology search process [14], [15]. Dmc1 strand exchange activity is sufficient for catalyzing D-loop formation during meiosis, although the presence of the Rad51 protein is necessary to regulate the activity of Dmc1 [16]–[19]. Coordinated action of the two RecA homologs is necessary for inter-homolog recombination bias [18], [19]. The assembly of Rad51 depends on Rad52, the Rad55-Rad57 complex and PCSS (Psy3-Csm2-Shu1-Shu2)/Shu complex as well as the single-stranded DNA binding protein RPA (Replication protein-A) [17], [20]–[22], whereas Dmc1 assembly depends on Mei5-Sae3 and Rad51 [19], [23]-[26]. Strand invasion by Rad51 and Dmc1 is facilitated by two Swi2/Snf2 DNA translocases, Rad54 and Tid1/Rdh54 [27]–[29].

Meiotic CO formation is also facilitated by a group of proteins called ZMM (Zip, Mer, Msh) or SIC (synaptic initiation complex), hereafter referred to as ZMM [13], [30]–[32]. ZMM proteins include Zip1, Zip2, Zip3, Mer3, Msh4, Msh5, Spo22/Zip4 and Spo16. Among these, Msh4 and Msh5 are MutS homologs [33]–[35]. The assembly of ZMM foci depends on DSB formation and processing [30]–[32], [36]. ZMM proteins also coordinate the formation of the synaptonemal complex (SC), a ladder-like structure consisting of two chromosomal axes flanking a central region, with the recombination. Zip1 is a component of the central region of the SC [37], [38]. Although, in the yeast, recombination promotes chromosome synapsis, chromosome synapsis is thought to control the processing of recombination intermediates between homologous chromosomes [39].

During meiosis, DNA damage checkpoint proteins play an important role in the cellular response to DNA damage as well as in DNA repair [40]. In the mitotic DNA damage checkpoint pathway of *Saccharomyces cerevisiae*, the Rad24-RFC (Rad17-RFC in other organisms) clamp-loader complex promotes the assembly of the Ddc1-Rad17-Mec3 clamp at the junction of tailed DNAs and activates downstream events [41], [42]. The Ddc1-Rad17-Mec3 clamp is referred as to the “9-1-1” (Rad9-Rad1-Hus1) complex in other organisms. In parallel, Mec1/ATR kinase is recruited to and activated on RPA-coated single-stranded DNAs in a Ddc2/ATRIP-dependent manner [43]. In meiosis, as in mitosis, checkpoint protein loading at the sites of recombination intermediates can induce delay in the entry into meiosis I [44]. In addition to providing a means to block meiotic progression when recombination is incomplete, the checkpoint proteins work directly in meiotic recombination [45]–[47]. For example, checkpoint proteins such as Rad17, Rad24 and Mec1 restrict the use of sister chromatids or repeated sequences at non-allelic (ectopic) sites as recombination partners [45], [47]. A direct role for checkpoint proteins in the recombination process is also suggested by the reduced rate of recombination in mutants lacking checkpoint function [46]. Consistent with this, over-expression of *RAD51* or *RAD54* can suppress the meiotic defects of checkpoint mutants as well as mitotic DNA damage sensitivity [46]. Moreover, the checkpoint clamp loader and clamp promotes CO formation by recruiting ZMM proteins to the chromosomes (Our unpublished results).

Previous genetic analyses showed that ectopic recombination occurs at frequencies similar to allelic recombination [48]–[52]. These studies showed that ectopic recombination between closely linked loci occurs more frequently than between dispersed loci. [48], [50], [51]. However, a genetic pathway(s) defining meiotic ectopic recombination (or NAHR) largely remains unknown. Genetic analysis, which requires viable gametes, is not applicable to mutants with reduced spore viabilities. The development of a physical assay for monitoring ectopic recombination has made it possible to dissect the pathway(s) that create these events and has revealed a critical role for DNA damage checkpoint genes in this process [45]. In this paper, we describe relationships among *RAD51*, *DMC1*, *ZIP1* and *RAD24*, that represent different classes of recombination genes, in the suppression of ectopic meiotic recombination. Our results provide evidence for two types of ectopic recombination: one that occurs in wild-type cells and a second that occurs predominantly when the checkpoint pathway is inactivated. We found that *RAD51* suppresses ectopic recombination, whereas *RAD52* is critical for it to occur. Moreover, chromosome synapsis did not appear to be involved in the suppression of ectopic recombination.

## Results

### The checkpoint clamp loader *RAD24* works in a common pathway with *RAD51* to suppress ectopic recombination

**Figure 1:**
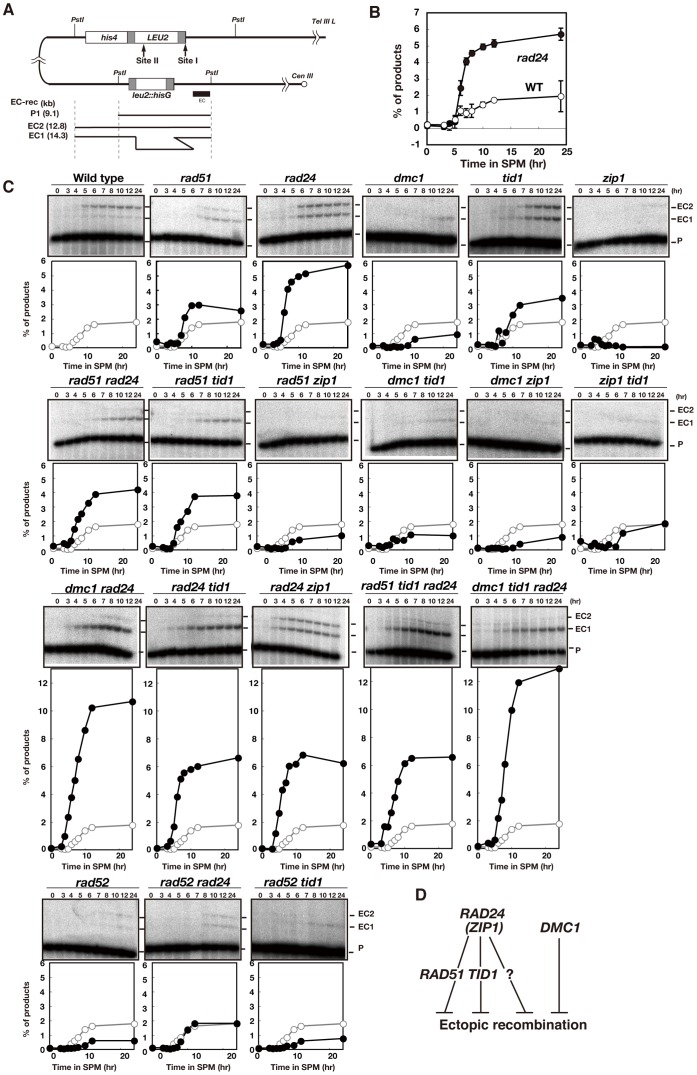
Meiotic ectopic recombination in various mutants. (A) Schematic representation of the *HIS4-LEU2* and *leu2::hisG* loci. Homologous regions between the *HIS4-LEU2* and *leu2::hisG* loci for ectopic recombination are shown in gray boxes. (B) Ectopic recombination in wild-type (WT) and *rad24* mutant cells was analyzed by Southern blotting. Genomic DNA was digested with *Pst*I. The percentage of ectopic recombination products (i.e., the relative amount of EC1 and EC2 bands) is shown. Error bars were obtained from three independent time courses. The error bars represent standard deviations (n = 3). Wild type, open circles; *rad24* mutant, closed circles. (C) Ectopic recombination in various mutants was analyzed by Southern blotting. Quantification of products as in B is shown. For each strain, at least two independent time courses were performed. Representative data are presented for each mutant strain (closed circles); wild-type data (open circles) are described in each blot for comparison. (D) A model overview of pathways for the suppression of ectopic recombination. The “?” gene is a putative gene which may suppress ectopic recombination.

Previous studies by Bishop and colleagues showed that checkpoint mutants such as *rad17*, *rad24*, and *mec1* increase ectopic recombination and that, when combined with *dmc1* mutation, the mutants also show synergestic increase of ectopic recombination [45]. In current studies, we extended these observations by studying the effect of different combinations of mutations in genes involved in meiotic recombination, DNA damage response as well as chromosome synapsis. A genetic interaction occurs between DNA damage checkpoint genes and *RAD51*, both in mitosis and meiosis [46]. We followed recombination products/intermediates in a physical assay using the recombination hot-spot *HIS4-LEU2* [53]. First, we measured products formed by ectopic recombination between the *HIS4-LEU2* and *leu2::hisG* locus on chromosome *III* (Figure 1A).

In the wild type, ectopic recombination products occurred at a frequency of 1.8% (Figure 1B and 1C). These recombination events were largely dependent on the meiosis-specific *recA* homolog *DMC1* (Figure 1C). The frequency of ectopic products increased to 5.8% in the *rad24* checkpoint mutant. These findings agree with a previous study [45]. Further, we found that a *rad51* mutant also showed a modest increase in ectopic recombination to 3.0% (Figure 1C), suggesting a role for *RAD51* in the suppression of ectopic recombination.

We also examined the relationship between checkpoint genes and the two RecA homologs *RAD51* and *DMC1* in ectopic recombination. The *rad51 rad24* double mutant exhibited an intermediate level of ectopic recombination as compared with that of *rad51* and *rad24* single mutants (Figure 1C). Given that the level of ectopic products in the *rad51* mutant was lower than that in the *rad24* single mutant or the *rad24 rad51* double mutant, the role of *RAD24* in suppression of ectopic recombination has both *RAD51*-dependent and *RAD51*-independent components. In contrast, in the *dmc1 rad24* double mutant there is a dramatic increase in ectopic recombination, up to 10.5% as shown previously [45], indicating that *DMC1,* unlike *RAD51*, can suppress ectopic events in the absence of *RAD24*.

### *TID1/RDH54* suppresses ectopic recombination

The DNA translocase Tid1/Rdh54 is required for normal meiotic recombination [54]. Tid1/Rdh54 promotes the recombination activity of both Rad51 and Dmc1 [29], [55]. When ectopic recombination was examined in the *tid1/rdh54* null mutant, there was a modest increase in ectopic products (∼2-fold as compared with wild type), similar to that of *rad51* (Figure 1C). Combining *rad51* and *tid1/rdh54* resulted in a higher level of ectopic products (1.3-fold) than that seen in the single mutants, suggesting differential contributions of *RAD51* and *TID1/RDH54* to suppression of ectopic recombination. Similar levels of ectopic recombination were observed in the *rad24* single, *tid1/rdh54 rad24* double and *rad51 tid1/rdh54 rad24* triple mutants. Thus, it appears that *rad24* suppression of ectopic recombination involves two additive components, a *RAD51*-dependent one and a *TID1/RDH54*-dependent one, which are partially overlapping functions. It should be noted that the level of ectopic recombination products in *rad24* is somewhat higher than that in *rad51 tid1/rdh54*, suggesting that there may be a third pathway of suppression of ectopic recombination mediated by *RAD24* (Figure 1D). These data suggest cooperation of *RAD24*, *RAD51* and *TID1/RDH54* in normal suppression of ectopic recombination. As in the case of the *rad24* single mutant, introduction of *dmc1* to the *tid1/rdh54 rad24* double mutant increases the frequency of ectopic recombination from 6% to ∼12%. This reinforces the idea that *DMC1*, not *RAD51* or its partner *TID1/RDH54*, plays a critical role in blocking ectopic events in the absence of *RAD24*.

### *RAD52* promotes ectopic recombination

Ectopic events occur even in the absence of the RecA homologs *RAD51* or *DMC1* (Figure 1C). Given that *RAD52* can promote recombination in the absence of *RAD51* in mitosis [56], [57], we also examined the role of *RAD52* in ectopic recombination. The *rad52* single mutant decreased ectopic recombination relative to that of wild type (2.7-fold; Figure 1C). Moreover, the combination of the *rad24* or the *tid1/rdh54* mutations with the *rad52* mutation reduced ectopic recombinant formation as compared with that of the respective single mutants by 2.9- and 7.5-fold, respectively. Therefore, *RAD52* plays a critical role in intra-chromosomal ectopic recombination in meiosis. However, the *rad52* mutation did not completely abolish ectopic recombination in any of the three backgrounds, indicating that some ectopic events occur independently of *RAD52* function. Therefore, most but not all ectopic events during meiosis depend on *RAD52*.

### A *ZMM* gene, *ZIP1*, is required for ectopic recombination in wild-type, *rad51* and *tid1/rdh54* cells

*ZMM* genes, including *ZIP1*, promote CO formation as well as chromosome synapsis [13]. Like all members of the *ZMM* group, the *zip1* mutant displays reduced CO levels, and increased NCO levels [13], [37], [38]. Without *ZIP1*, paired axial elements do not synapse [38]. We hypothesized that ectopic recombination levels might be restricted by assembly of the SC pairing interactions and therefore elevated in the *zip1* mutant because of its synapsis defect. Contrary to this prediction, ectopic recombinant levels were markedly reduced to almost undetectable levels in the *zip1* mutant (7.3-fold; Figure 1C), indicating that rather than repressing ectopic recombination, *ZIP1* is required for it to occur. The negative effect of the *zip1* mutation on ectopic recombination levels was largely epistatic to the positive effects of *rad51* and *tid1/rdh54* mutations; the *rad51 zip1* and *tid1/rdh54 zip1* double mutants showed 4.4- and 3.8-fold reductions, respectively, in ectopic products relative to levels in the respective single mutants (Figure 1C). Thus, *ZIP1* appears to play a critical role in the formation of ectopic products in the wild-type contexts as well as in *rad51* and *tid1/rdh54* mutant contexts.

### *rad24* is epistatic to *zip1* in meiotic recombination

In contrast to the situation in the wild type or in *rad51* and *tid1/rdh54* single mutants, addition of a *zip1* mutation in the *rad24* mutant did not alter the abnormally high levels of ectopic products (Figure 1C). This result indicates that there are two distinct pathways for ectopic recombination, one that predominates in wild-type cells and depends on *ZIP1*, and a second pathway seen in the *rad24* mutant that is *ZIP1*-independent (Figure 1D). This is consistent with the recent finding that the Rad24 clamp loader is necessary for efficient loading of ZMM proteins onto meiotic chromosomes (MS & AS, unpublished results).

### *RAD51* and *RAD24* suppress resection of DSB ends

**Figure 2:**
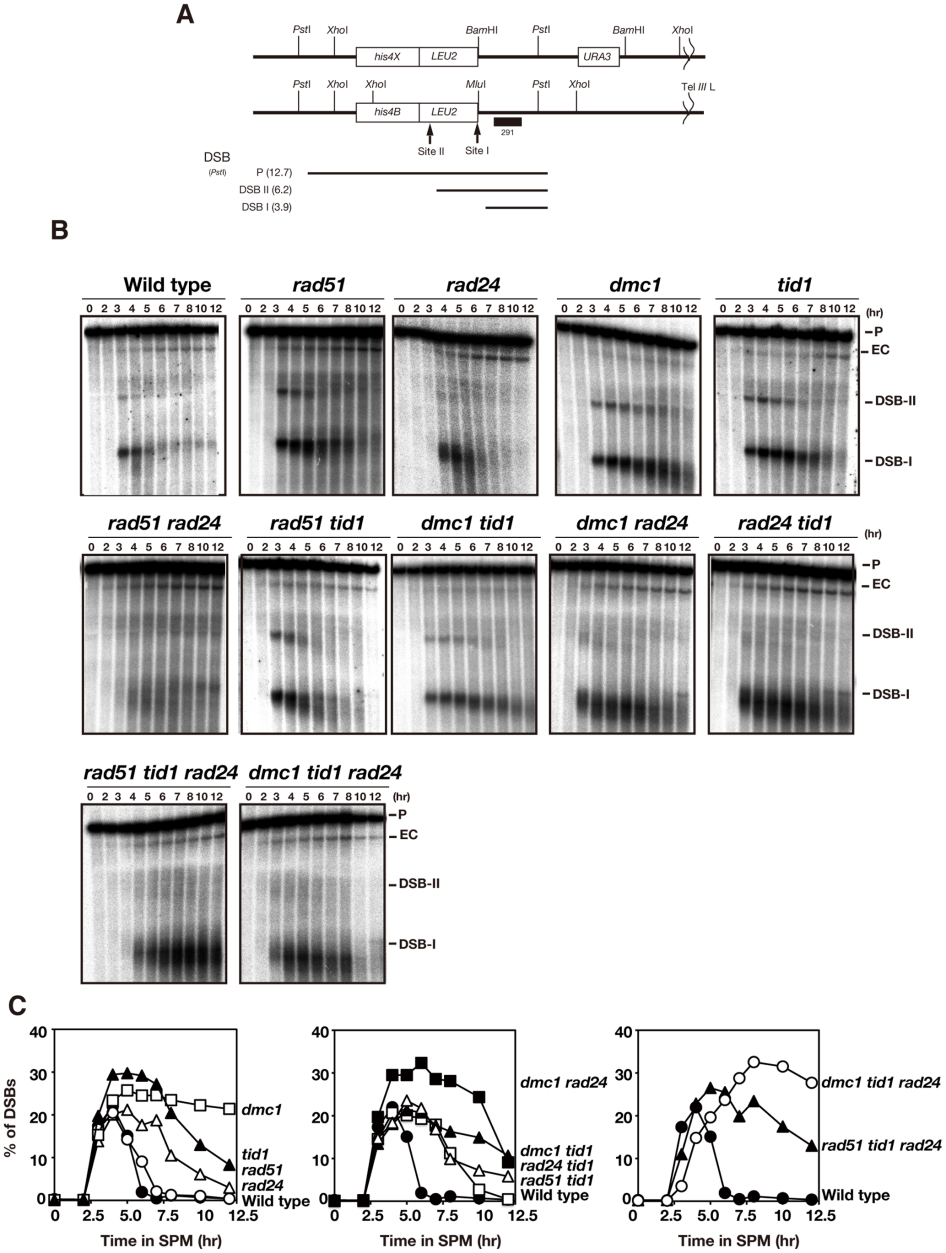
DSB repair in various mutants. (A) Schematic representation of *HIS4-LEU2* locus for DSB detection. (B) DSB repair in wild-type and various mutant cells was analyzed by Southern blotting. Genomic DNAs were digested with *Pst*I. For each strain, at least two independent time courses were performed. Representative data are shown for each strain. (C) Quantification of DSB at site I (B). The quantification of DSBs in the *rad51 rad24* mutant is not shown due to smear nature of DSB bands. Left graph, wild type, closed circles; *rad24*, open circles; *rad51*, open triangles; *tid1*, closed triangles; *dmc1*, open rectangles. Middle graph, wild type, closed circles; *rad24 tid1*, open triangles; *dmc1 tid1*, closed triangles; *rad51 tid1*, open rectangles; *rad24 dmc1*, closed rectangles. Right graph, wild type, closed circles; *dmc1 tid1 rad24*, open circles; *rad51 tid1 rad24*, closed triangle.

We also monitored DSB repair at the *HIS4-LEU2* locus in above mutants (Figure 2A). All single, double and triple mutants showed delayed disappearance of DSBs (Figure 2B and 2C), indicating a defect in the repair of DSBs during meiosis. Most of the mutant combinations, particularly ones with the *rad24* mutation, also showed elevated resection of the DSB ends relative to the wild type. The *rad51 rad24* double mutant, in particular, exhibited particularly diffuse bands in the DSB regions, which are indicative of extensive nucleolytic degradation (Figure 2B). This suggests that *RAD51* and *RAD24* have redundant roles in the protection of DSB ends from nuclease attack during meiosis. A similar protective role for *rad24* during mitosis has been reported [58].

## Discussion

**Figure 3:**
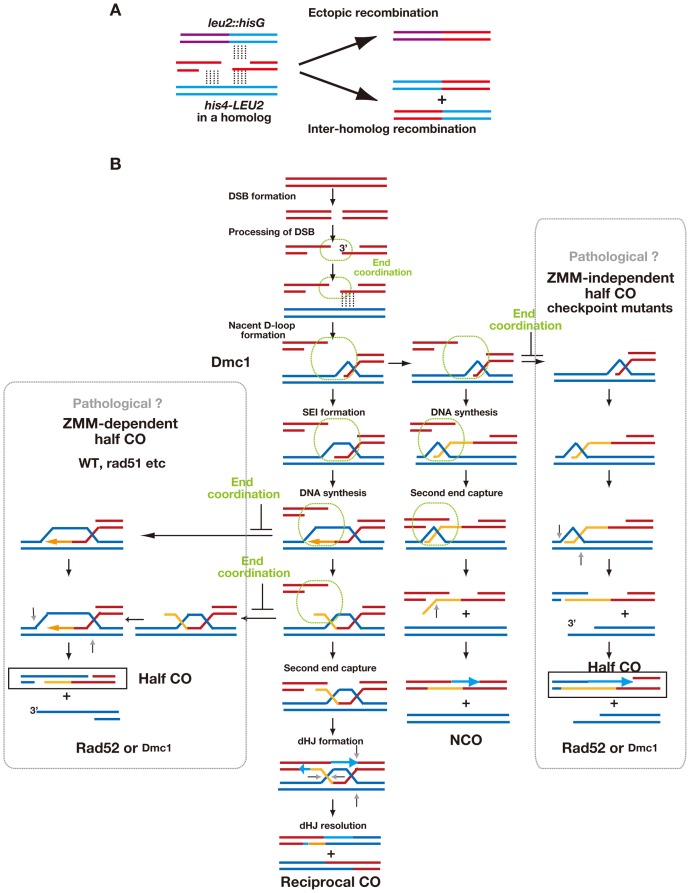
A model of meiotic recombination with pathological pathways. (A) Only one side of DSB I in the *HIS4-LEU2* locus has homology to *leu2::hisG*, which may lead to half-COs (top). A heterologous region of the *leu2::hisG* to *LEU2* (left) is shown in purple. This is distinct from a canonical reciprocal CO pathway (bottom). (B) Two potential pathological pathways for ectopic recombination, which result in the formation of half-COs. Parental DNAs are shown in red or blue. Newly-synthesized strand is in orange. End-coordination is shown in green.

Meiotic recombination can produce two distinct products, COs and NCOs. These products are formed through different branches of the recombination pathway after the formation of DSBs (Figure 3). Meiotic COs are predominantly reciprocal between homologous chromosomes and are promoted by ZMM proteins. ZMM -dependent recombination intermediates appear to be critical for reciprocal CO formation, with coordinated direction of the two DSB ends to the same target for exchange [13]. By analyzing intra-chromosomal ectopic recombination, we were able to describe a novel pathway(s) for the formation of half-CO products. The exchange between *LEU2* of *HIS4-LEU2* and *leu2::hisG*, which was initiated at DSB site I [45], is forced to be non-reciprocal, as the homology is limited to one end of the DSB (*LEU2* side; Figure 3A). Wild-type cells showed very low levels of ectopic recombination, whereas some mutants showed increased levels of these half-CO events. This suggests that targeting of the second DSB end to the same site as the first is regulated. A simple mechanism for end-coordination would be physical linkage of the two ends. Alternatively, interaction of the second end with the target could be indirectly regulated by interactions of the first end. For example, there might be a negative feedback mechanism to guarantee that both ends go to the same target DNA. This could occur by regulated asymmetric loading of recombination enzymes such that only one end of a DSB is endowed with strand invasion activity [9], [25], [28] or by specific disruption of interactions in which opposite DSB ends engage different targets [8], [59]. A non-exclusive alternative is that ectopic recombination between *HIS4-LEU2* and *leu2::hisG* might be repressed because of structural constraints coupled with end coordination. For example, partner ends might be constrained to a local segment of the axial element [60], [61].

One interesting observation in this study is that ectopic recombination in some strains largely depended on the *ZMM* gene *ZIP1*. This *ZIP1*-dependent ectopic recombination seems to operate in wild-type, *rad51*, *tid1/rdh54* and *dmc1* cells. It is likely that *ZIP1*-dependent ectopic recombination is a pathological outcome that occurs when the normal end coordination mechanism fails (left pathway in Figure 3B). Because *ZMM* genes play a critical role in the formation of SEIs and dHJs [7], [13], it is likely that half-CO products in this pathway are formed via single-end interactions that yield SEIs or dHJs as intermediates.

In addition, there is a second pathway by which half-COs can occur during meiosis (pathway on the right in Figure 3B) that appears to be activated when checkpoint signaling is defective. Unlike the case in wild-type cells, ectopic recombinant formation in the checkpoint mutant, *rad24*, did not require ZMM function. Given that the checkpoint mutation was epistatic to the *zip1* mutation with respect to meiotic recombination, this *ZMM*-independent ectopic recombination appears to reflect a pathway branch based on an earlier decision point than the normal *ZMM*-dependent recombination. In wild-type cells, the *ZMM*-dependent CO pathway follows the early CO/NCO decision with NCO pathway is branched as a default [7], [10]. Thus, it is likely that *ZMM*-independent ectopic products are a pathological derivative of the normal NCO pathway. Again we propose that this pathway is suppressed in the wild type by end-coordination functions. Our results strongly suggest that DSB end-coordination is key to the progression of the recombination complex to intermediates that are designated to become COs via the *ZMM* pathway.

Many of the requirements for ectopic recombination are different from those for allelic recombination. Two *recA* homologs, *RAD51* and *DMC1*, are critical for inter-homolog recombination. The data in the paper show that *RAD51* suppresses ectopic recombination in an otherwise wild-type strain whereas *DMC1* only suppresses ectopic recombination in the *rad24* background. Strand invasion in ectopic recombination seems to be carried out by Rad52 as well as Dmc1. Consistent with this, Rad52 catalyzes D-loop formation *in vitro* [62]. The role of Rad52 in half-CO formation might be similar to that in break-induced replication during mitosis [56]. Among proteins that suppress ectopic recombination, checkpoint proteins appear to play the most important role. The role of checkpoint proteins in the suppression does not appear to be related to their role in CO formation through the recruitment of ZMM proteins. Rather, the ability of checkpoint proteins to collaborate with Rad51/Rad54 for DNA repair processes [46] might be relevant.

It is widely believed that chromosome synapsis promotes recombination between homologous chromosomes. Earlier study on ectopic recombination suggests that interhomolog interactions restrict ectopic recombination [52]. We found that the *zip1* mutant, which is defective in chromosome synapsis, reduces ectopic recombination. This suggests that chromosome synapsis may not be involved in the early steps of recombination, such as D-loop formation. Instead, Zip1 appears to be involved in stabilizing later recombination intermediates to promote CO.

Our physical study here as well as previous genetic studies on NAHR provided new insight on the ectopic recombination in meiosis [48]–[52]. However, it is important to point out that these studies used assays for ectopic recombination between sequences where the extent of homology is severely limited by nearby flanking heterology. We need to develop an assay to study ectopic recombination between sequences with long homology; e.g. recombination between sequences with more than 10kb homology on different chromosomal locations.

Our results showed that defects in the canonical meiotic recombination pathways lead to increased NAHR, which would presumably result in chromosome instability in gametes [4]. Therefore, genes involved in meiotic recombination may be a risk factor for genome instability.

## Materials and Methods

### Strains

All strains described here are derivatives of the *S. cerevisiae* SK1 diploid strain NKY1551 (*MATα/MAT* ***a*** *, lys2/”, ura3/”, leu2::hisG/”, his4X-LEU2-URA3/his4B-LEU2, arg4-nsp/arg4-bgl*). The genotypes of each strain used in this study are described in Table 1. The *tid1/rdh54* null alleles were constructed by PCR-mediated gene disruption using *KanMX6* genes as described [63].

**Table 1:** Strain list.

Strain Number	Genotypes
NKY1551	*MATα*/***a*** *, ho::LYS2/”, lys2/”, ura3/”, leu2::hisG/”, his4X-LEU2(BamHI)-URA3/his4B-LEU2(MluI), arg4-nsp/arg4-bgl*
MSY2746	NKY1551 with *rad51::hisG-URA3-hisG*
MSY717	NKY1551 with *rad24::LEU2*
MSY2237	NKY1551 with *dmc1::LEU2*
MSY1712	NKY1551 with *tid1/rdh54::KanMX6*
MSY2820	NKY1551 with *zip1::LEU2*
MSY2082	NKY1551 with *rad51::hisG-URA3-hisG, rad24::LEU2*
MSY2069	NKY1551 with *rad51::hisG-URA3-hisG, tid1/rdh54::KanMX6*
MSY2712	NKY1551 with *rad51::hisG-URA3-hisG, zip1::LEU2*
MSY219	NKY1551 with *dmc1::LEU2, tid1/rdh54::KanMX6*
MSY2719	NKY1551 with *dmc1::LEU2, zip1::LEU2*
MSY2135	NKY1551 with *tid1::KanMX6, zip1::LEU2*
MSY2096	NKY1551 with *dmc1::LEU2, rad24::LEU2*
MSY2103	NKY1551 with *rad24::LEU2, tid1/rdh54::KanMX6*
MSY2168	NKY1551 with *rad24::LEU2, zip1::LEU2*
MSY2122	NKY1551 with *rad51::hisG-URA3-hisG, tid1/rdh54::KanMX6, rad24::LEU2*
MSY2137	NKY1551 with *dmc1::LEU2, tid1/rdh54::KanMX6, rad24::LEU2*
MSY2777	NKY1551 with *rad52::hisG-URA3-hisG*
MSY2822	NKY1551 with *rad52::hisG-URA3-hisG, rad24::LEU2*
MSY2825	NKY1551 with *rad52::hisG-URA3-hisG, tid1/rdh54::KanMX6*

### Analyses of meiotic recombination

Time course analyses of DNA events in meiosis and cell cycle progression were performed as described [29]. Ectopic recombination was analyzed as described [45]. Blots were scanned using a PhosphoImager, BAS3,000 (Fuji film) and quantified using ImageQuant (GE Healthcare) software. Ectopic recombination and DSB repair were analyzed more than twice and representative results are shown.
